# Structural Basis of Enzymatic Activity for the Ferulic Acid Decarboxylase (FADase) from *Enterobacter* sp. Px6-4

**DOI:** 10.1371/journal.pone.0016262

**Published:** 2011-01-21

**Authors:** Wen Gu, Jinkui Yang, Zhiyong Lou, Lianming Liang, Yuna Sun, Jingwen Huang, Xuemei Li, Yi Cao, Zhaohui Meng, Ke-Qin Zhang

**Affiliations:** 1 Laboratory for Conservation and Utilization of Bio-Resources, Key Laboratory for Microbial Resources of the Ministry of Education, Yunnan University, Kunming, China; 2 Structural Biology Laboratory, Tsinghua University, Beijing, China; 3 National Laboratory of Macromolecules, Institute of Biophysics, Chinese Academy of Science, Beijing, China; 4 Yunnan Academy of Tobacco Science, Kunming, China; 5 Guizhou Tobacco Research Institute, Guiyang, China; 6 Laboratory of Molecular Cardiology, Department of Cardiology, The First Affiliated Hospital of Kunming Medical College, Kunming, China; Griffith University, Australia

## Abstract

Microbial ferulic acid decarboxylase (FADase) catalyzes the transformation of ferulic acid to 4-hydroxy-3-methoxystyrene (4-vinylguaiacol) via non-oxidative decarboxylation. Here we report the crystal structures of the *Enterobacter* sp. Px6-4 FADase and the enzyme in complex with substrate analogues. Our analyses revealed that FADase possessed a half-opened bottom β-barrel with the catalytic pocket located between the middle of the core β-barrel and the helical bottom. Its structure shared a high degree of similarity with members of the phenolic acid decarboxylase (PAD) superfamily. Structural analysis revealed that FADase catalyzed reactions by an “open-closed” mechanism involving a pocket of 8×8×15 Å dimension on the surface of the enzyme. The active pocket could directly contact the solvent and allow the substrate to enter when induced by substrate analogues. Site-directed mutagenesis showed that the E134A mutation decreased the enzyme activity by more than 60%, and Y21A and Y27A mutations abolished the enzyme activity completely. The combined structural and mutagenesis results suggest that during decarboxylation of ferulic acid by FADase, Trp25 and Tyr27 are required for the entering and proper orientation of the substrate while Glu134 and Asn23 participate in proton transfer.

## Introduction

Phenolic acids, mainly *p*-coumaric and ferulic acids, are covalently bound to polysaccharides in cell walls of higher plants. These acids are essential for the growth and reproduction of plants, and are commonly produced as part of the defense against pathogen infection at injured sites in plants [1]. Ferulic acid〔 [3-(4-hydroxy-3-methoxyphenyl)-2-propenoic acid〕] is an abundant hydroxycinnamic acid in the plants and can be transformed by microorganisms into valuable aromatic compounds such as vinylguaiacol and vanillin [2]. In plants, this acid may exist in a free form or be covalently linked to lignin and other polymers in the cell wall [3]. Because of its natural abundance and environmental safety profile, ferulic acid is commonly used as a substrate for the production of natural vanillin via biotransformation [2]. Vanillin (4-hydroxy-3-methoxybenzaldehyde) is widely used as a nature flavoring compound in foods, beverages, perfumes, and other consumer products [4], [5]. Globally, more than 12,000 tons of vanillin is produced each year. Even though less than 1% of the vanillin is extracted from *Vanilla* orchid pods, the value of naturally extracted vanillin is much higher than that of the artificially synthesized vanillin [6], [7]. Strong market demand for natural vanillin has spawned efforts to produce it by microbial transformation from natural substrates, including phenolic stibenes [8], eugenol [9], [10], and ferulic acid [7], [11].

Several plants, fungi, bacteria, actinomycetes, and microalgae have been reported capable of transforming ferulic acid into vanillin and other related metabolites [5], [12]. Four major pathways of ferulic acid transformation can be distinguished with respect to the initial reaction: (i) non-oxidative decarboxylation; (ii) side chain reduction; (iii) coenzyme-A-independent deacetylation, and (iv) coenzyme-A-dependent deacetylation [2], [3], [5]. Ferulic acid decarboxylase (FADase) catalyzes the non-oxidative decarboxylation of ferulic acid to produce 4-vinylguaiacol. Non-oxidative decarboxylation of ferulic acid by FADase has been discovered in many fungi and yeasts [13], [14], [15], [16], [17] as well as in some bacteria [18], [19], [20], [21], [22]. Recently, the crystal structures of two *p-*coumaric acid decarboxylase (PDC, an enzyme similar to FADase) were reported, one from *Lactobacillus plantarum* (PDB code: 2W2A) and the other from *Bacillus subtilis* (PDB code: 2P8G) [14], [23], However, the precise catalytic mechanism of FADase remains largely unknown. The crystallization and co-crystallization of FADase in complex with inhibitor or substrate analogues is necessary for elucidating the catalytic mechanism of FADase.

We recently reported that the bacterium *Enterobacter* sp. Px6-4 isolated from vanilla roots could utilize ferulic acid as the sole carbon source to produce vanillin by transforming ferulic acid to 4-vinylguaiacol via the non-oxidative decarboxylation [24]. To understand the detailed mechanism of action of FADase from *Enterobacter* sp. Px6-4, we cloned and expressed the FADase gene in *E. coli* BL21 (DE3) and solved the crystal structures of FADase and FADase in complex with a substrate analog sodium ferulate. Analyses of the crystal structure and mutagenesis studies revealed an “open-closed” pattern of FADase catalysis. The combined structural and mutagenesis results enabled us to propose the catalytic mechanism of FADase.

## Materials and Methods

### Strains and vectors

*Enterobacter* sp. Px6-4 was isolated from a vanilla root and deposited in the China General Microbiological Culture Collection Center (CGMCC 1999). *Escherichia coli* strains DH5α and *E. coli* BL21 were used as host cells for the transformation and propagation of plasmids harboring desired DNA fragments. All bacteria were grown in Luria-Bertani (LB) medium at 37°C.

Vectors pMD18-T (Takara, Japan) and pET-28a (+) (Novagen, Germany) were used for TA-cloning and gene expression, respectively.

### Expression and purification of FADase expressed in *E. coli* BL21

Based on the sequence deposited in GenBank (accession no. EU853825), the FADase gene was amplified using primers PX1e containing an *Eco*RI restriction site (5′-AGGGAATTCATGAACACCTTCGACAAACA-3′) and PX2e containing a *Hin*dIII restriction site (5′-GGCAAGCTTGCGCTTATTTTA AATTATCAGG-3′). The amplified product was inserted into plasmid pET-28a. The recombinant vector pET-28a (+)/*fad* was transformed into *E. coli* BL21 (DE3) following the user's protocol (Novagen, Germany). Under 0.2 mM IPTG induction at 37°C overnight, FADase was highly expressed as a soluble protein in *E. coli* BL21 (DE3). Purification of the FADase protein was carried out through a 2-ml nickel-nitrilotriacetate column (Qiagen, German), a Resource Q column (Amersham, Sweden), and a HiPrep 16/10 Phenyl FF (high sub) column (Amersham, Sweden). The purified protein was confirmed by denaturing SDS-PAGE.

### Crystallization

Crystallization was performed at 290 K using the hanging-drop vapor-diffusion technique. A series of crystallization grids were prepared by mixing 10 mg/ml apo-enzyme in 20 mM Tris-HCl, pH7.0 with equal volumes of reservoir solution containing 0.1 M HEPES pH7.3, 27% w/v PEG10000. Crystals of FADase complexed with substrate analog sodium ferulate were obtained by adding an equal volume of sodium ferulate (7 mM) into the FADase crystal drop and then incubated overnight. The initial crystals were typically macroscopically twinned. Single, larger crystals suitable for data collection were eventually obtained and were soaked in a cryoprotectant consisting of reservoir solution and 20% (v/v) glycerol. Crystals were flash-frozen in liquid nitrogen and then transferred into a dry nitrogen stream at 100 K for X-ray data collection.

### Data collection and structure determination

The data was collected for native FADase at 100 K using a Mar165 CCD detector on beamline 3W1A at the Beijing Synchrotron Radiation Facility. Data were processed and scaled using the HKL2000 package [25]. Crystals were found belonging to the space group *P2_1_*, with two monomers in the asymmetric unit. The Matthews coefficient was calculated as 2.6, corresponding to 46% solvent content [26]. Initial phases were obtained by molecular replacement with PHASER [27] using the crystal structure of phenolic acid decarboxylase (PDB code: 2P8G) as the searching model. The structure of FADase complexed with sodium ferulate was also solved using PHASER with native FADase structure as the searching model. The bound sodium ferulate was built based on the *Fo-Fc* difference electron density map contoured at +2.5 sigma. The final manual rebuilding and refinement were performed in COOT [28] and Refmac5 [29] based on the *2Fo-Fc* and *Fo-Fc* map. During the later stages of positional refinement, restraints were relaxed and a bulk solvent correction was applied under the guidance of *R_free_*. Model geometry was verified using the program PROCHECK [30]. Solvent molecules were located from stereochemically reasonable peaks in the σA-weighted *Fo-Fc* difference electron density map. Final refinement statistics are shown in Table 1. Figures were created using PYMOL [31]. Coordinates and structural factors have been deposited in the Protein Data Bank with accession codes 3NX1 (apo-FADase) and 3NX2 (complex).

**Table 1:** Data collection and refinement statistics.

Parameters	FADase Native	FADase complexed with sodium ferulate
Data collection statistics		
Cell parameters	*a* = 44.5 Å*b* = 88.7 Å*c* = 49.3 Åα = γ = 90°β = 102.2°	*a* = 43.3 Å*b* = 88.7 Å*c* = 49.0 Åα = γ = 90°β = 102.3°
Space group	*P2_1_*	*P2_1_*
Wavelength used (Å)	1.0000	1.5418
Resolution (Å)	50.0(2.5)^c^ – 2.4	50 (2.2) – 2.1
No. of all reflections	79,804	85,762
No. of unique reflections	12,722	23,508
Completeness (%)	93.0 (70.9)	95.0 (92.0)
Average I/σ(I)	6.3 (2.2)	7.7 (2.1)
R_merge_ a (%)	11.5 (33.0)	8.7 (43.0)
Refinement statistics		
No. of reflections used (σ(F) >0)	12,090	22,288
R_work_ b (%)	16.2	18.9
R_free_ b (%)	26.1	23.5
r.m.s.d. bond distance (Å)	0.011	0.008
r.m.s.d. bond angle (°)	1.532	1.451
Average B-factor, protein (Å^2^)	26.8	29.2
Average B-factor, ligand (Å^2^)		37.0
Ramachandran plot (excluding Pro & Gly)		
Res. in most favored regions	235 (83.0%)	250 (88.3%)
Res. in additionally allowed regions	45 (16.3%)	32 (11.3%)
Res. in generously allowed regions	2 (0.7%)	1 (0.4%)

a *R_merge_*  = Σ_h_Σ_l_ | I_ih_−<I_h_>|/Σ_h_Σ_I_ <I_h_>, where <I_h_> is the mean of the observations I_ih_ of reflection h.

b *R_work_*  =  Σ(||F_p_(obs)|−|F_p_(calc)||)/Σ|F_p_(obs)|; *R_free_*  = R factor for a selected subset (5%) of the reflections that was not included in prior refinement calculations.

c Numbers in parentheses are corresponding values for the highest resolution shell.

### Enzyme analysis and mutagenesis

The concentration of the FADase protein was determined by the method of Bradford [32] using bovine serum album as a standard. The effects of pH and temperature on enzyme activity were determined following a published method [33], using ferulic acid as substrate. The enzyme activity of FADase was determined under the condition of 50 mM sodium phosphate buffer (pH 4.0) and 2 mM of ferulic acid. The reaction mixture contained purified FADase and ferulic acid was incubated at 28°C and sampled at 1 minute intervals. The concentration of substrates and products was determined using HPLC [24]. All assays were repeated three times.

The mode of inhibition by substrate analog sodium ferulate was determined as follows. Inhibitor affinity was first estimated by determining IC_50_ values in a standard assay [34]. *Ki* values were then approximated by determining kinetic parameters (*Km*) in the presence of inhibitor at a concentration close to the IC_50_. Finally, the mode of action was determined by plotting the data as Lineweaver-Burk plots, and by fitting all data to the standard competitive inhibition equation [35].

Site-directed mutagenesis of the FADase gene was carried out using the MutanBEST Kit (TaKaRa, Japan). DNA sequences of the mutated gene fragments were confirmed using an ABI PRISM 3730 DNA Sequencer (Perkin–Elmer Applied Biosystems, USA). Mutant genes were cloned and over-expressed in *E. coli* BL21 as described for the wild-type FADase gene. Similarly, mutated FADase proteins were purified following those described for the wild-type protein.

## Results

### Overall structure of FADase

The full-length FADase (residues Met1-Lys168) encoding gene *fad* (EU853825) was amplified, cloned into vector pET-28a, and expressed in *E. coli* BL21 (DE3) using isopropyl β-D-1-thiogalactopyranoside (IPTG) as the inducer. The recombinant FADase was purified as a protein of 23-kDa.

The crystal structures were determined to 2.4 Å and 2.1 Å resolutions respectively for the pure FADase and FADase complexed with sodium ferulate. Data collection and refinement statistics of the structures are summarized in Table 1. There were two molecules in one asymmetric unit with a Matthews coefficient of 2.6 at 46% solvent content. The result was consistent with the dimerization state of FADase, which was first observed in gel-filtration chromatography and supported further by crosslinking with ethylene glycolbis (Figure S1). The results also suggested that the biologically active form for FADase was a homodimer.

**Figure 1:**
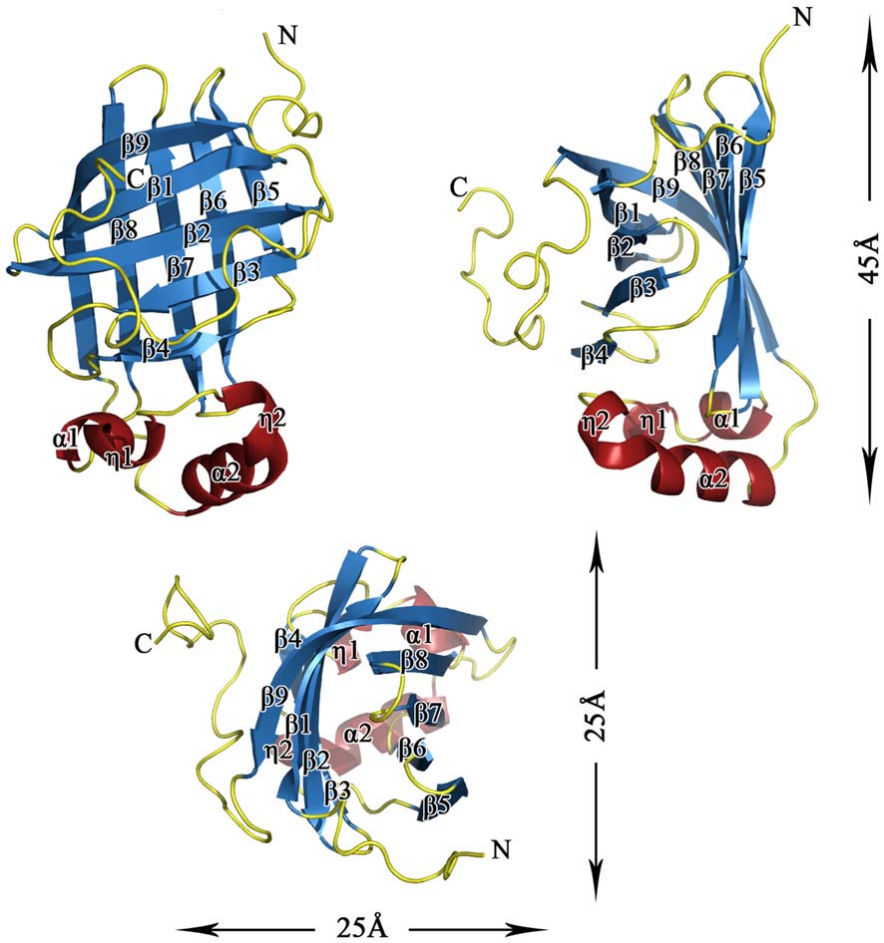
The monomer fold of FADase. The cylinder-shaped monomer of FADase with an overall dimension of 25×25×45 Å. The β-barrels are shown in blue, helices in red, and loops in yellow.

The cylinder-shaped monomer of FADase had an overall dimension of 25×25×45 Å and is composed of nine β-strands, two α-helices and two η-helices (Figure 1). The FADase molecule could be divided into three independent components: the core, a helical bottom, and a C terminal extension. The core was formed by anti-parallel β1 to β9, which together form an open-ended β-barrel. The helical bottom, composed of α1, α2 and η1, η2, was located at the bottom of the core β-barrel. The catalytic center was located between the core β-barrel and the helical bottom and was surrounded by α2, β8, β9, and the hair-pins between β1/2 and β3/4. The conformational change of the β hair-pins was related to substrate binding. The C terminal extension, which contained a long coil with η2 in the middle, was located behind β9.

### The substrate binding site

**Figure 2:**
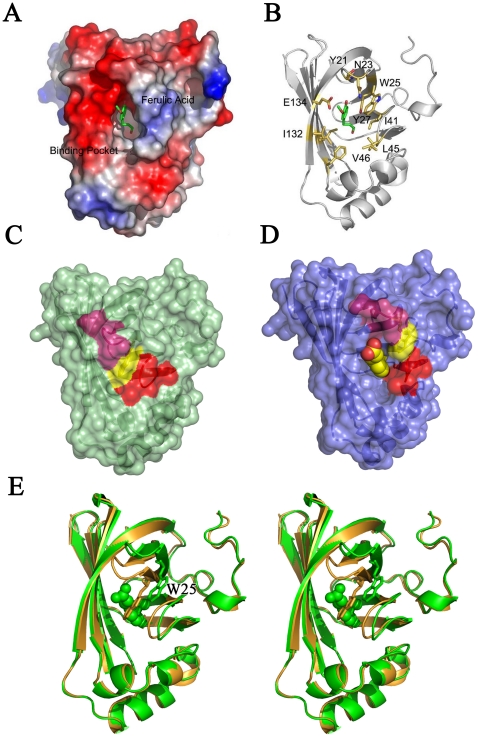
Composition of the substrate binding pocket. A. The potential surface of FADase complexed with sodium ferulate. The FADase molecule is represented by potential surface. B. FADase molecule is represented by white ribbon; the residues, which form binding pocket, are drawn as gold sticks. The bound sodium ferulate is shown as green sticks. C. The closed form of FADase binding pocket. D. The open form of FADase binding pocket. Two hair-pins, which act as lids of pocket, are labeled as warm pink and red; Trp25, which acts as a lock, is labeled as yellow. The substrate is marked as spheres. E. The stereo view of binding pocket. Substrate molecules are shown as spheres; the key residue W25 is shown as sticks and highlighted. The closed and open forms were colored as gold and green, respectively.

**Figure 3:**
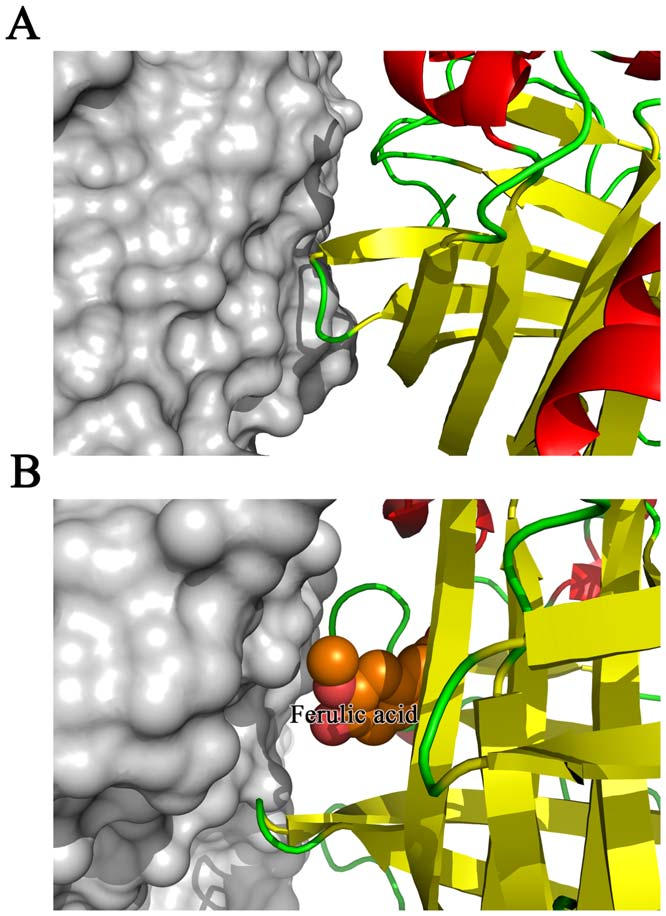
The sketch map of FADase's crystal packing. A. The closed form of the active pocket, shown as a cartoon, contacted with sideward FADase shown as a surface. B. The opened form of active pocket, shown as a cartoon, contacted with sideward FADase shown as a surface, and substrate was shown as spheres and labeled.

The crystal structure of FADase in complex with the substrate analog sodium ferulate revealed the substrate-binding pocket of FADase (Figure 2). Crystal packing in FADase crystals resulted in the substrate binding only to monomer A of the two monomers. The substrate-binding pocket is illustrated using monomer A (Figure 3).

**Figure 4:**
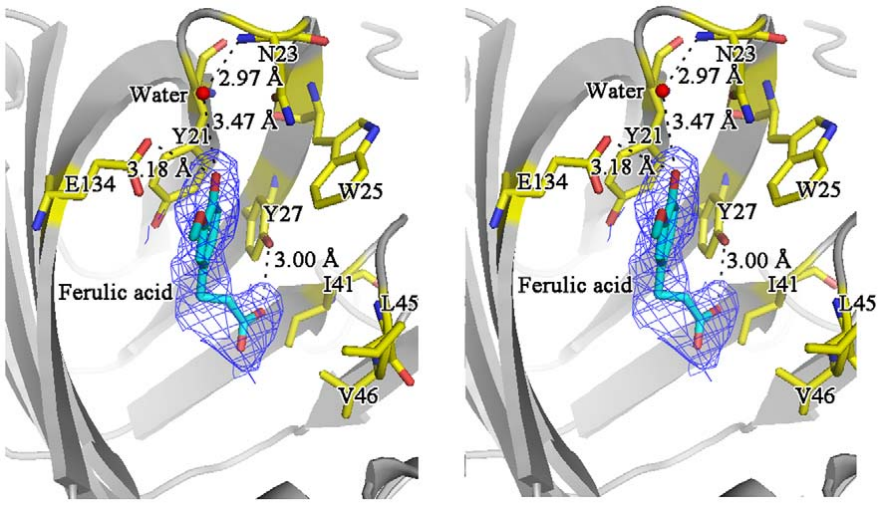
The complex of ferulic acid bound to FADase, as a stereo view. All key residues and ferulic acid are shown as gold sticks respectively, and FADase molecule is shown as white ribbon. The bound ferulic acid is covered with 2.5σ *Fo*-*Fc* map. Hydrogen bonds are shown as black dashed.

The substrate analog sodium ferulate was found binding to FADase in the pocket located between the core β-barrel and the helical bottom (Figure 2A, 2B). At the half-opened bottom, the residues from β8, β9 and hair-pins between β1/2, β3/4 constituted the hydrophobic substrate binding pocket (Figure 2A). Here, the hair-pins acted as two lids of the active site (Figure 2C, 2D) and we named them lid 1 and lid 2. Moreover, Trp25 acted as a lock in the interaction (Fig. 2C, D). In the closed form, the two hair-pins moved towards β9 by about 5 Å to seal the pocket and shield the substrate away from the catalytic center. In the open form, the two hair-pins moved away from each other to accept substrate into the pocket (Figure 2C, 2D). In the open form, the pocket had a dimension of 8×8×15 Å with the active center consisting residues Tyr21, Asn23, Trp25, Tyr27, Ile41, Leu45, Val46, Ile132 and Glu134. These residues formed the substrate-binding site and they belonged to either the aromatic R groups (Tyr21, Trp25 and Tyr27) or the aliphatic R groups (Asn23, Ile41, Leu45, Val46, Ile132 and Glu134). Aromatic R groups were placed at the active pocket's upper part, and aliphatic R groups were located at its lower part. There were three main residues related to substrate binding: Tyr27, Glu134, and Asn23. Tyr27 and Glu134 directly linked the substrate's carboxyl and hydroxyl groups through hydrogen bonding. The Tyr27- carboxyl and Glu134- hydroxyl bond distances were 3.00 Å and 3.18 Å, respectively. Asn23 indirectly linked the substrate's hydroxyl group by a water molecule. The bond distance between Asn23 and the water molecule was 2.97 Å, and between the water molecule and the substrate's hydroxyl group was 3.47 Å (Figure 4).

### Comparisons of FADase, PAD and PDC

**Figure 5:**
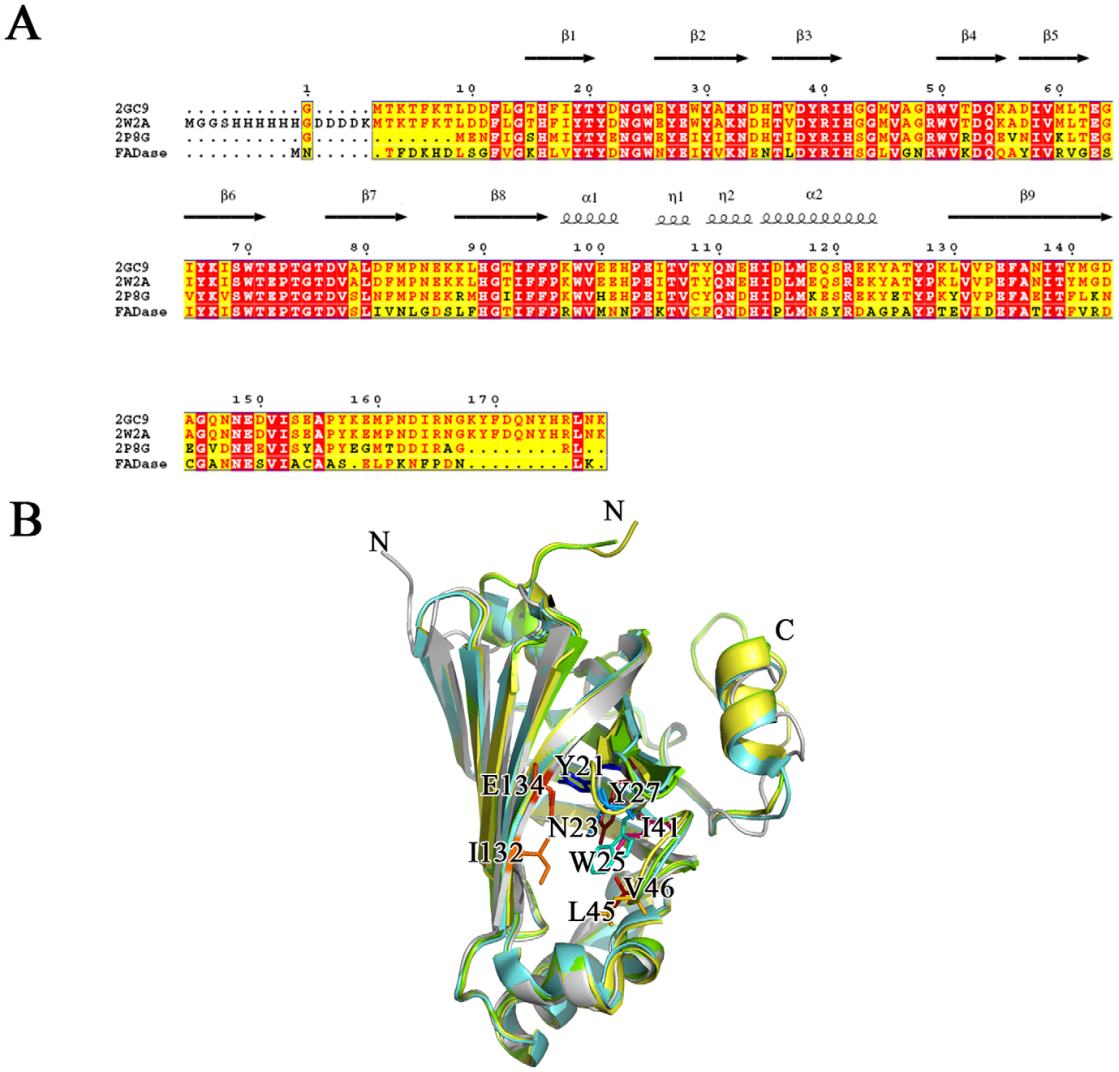
Sequence and structural similarity of FADase, PDC and PAD. A. The structure based sequence alignment of FADase, PDC and PAD. The secondary structures of FADase are labeled out. B. The structure superposition of FADase, PDC (PDB code: 2GC9 and 2W2A) and PAD (PDB code: 2P8G). These four structures are colored as white, yellow, green and blue respectively. Substrate binding sites of FADase are shown as sticks and labeled.

Amino acid sequence and structural similarity analysis suggested that PAD (PDB code: 2P8G, sequence identity of 55%) from *B. subtilis* and PDC (PDB code: 2GC9 and 2W2A, sequence identity of 54%) from *L. plantarum* [23] shared a high degree of sequence and structural similarity with FADase. Although substrates of the three enzymes are different, all the substrates belong to the hydroxycinnamic acid family. Moreover, PAD and PDC share structural similarity with FADase with Ca r.m.s.d of 0.584 Å (2W2A), 0.522 Å (2GC9) and 0.606 Å (2P8G), respectively. Consistent with the structural conservation, the residues forming the half-opened bottom of the catalytic center were highly conserved (Figure 5A). The differences among the three structures were mainly found in the random coil of the N-terminal and the C-terminal of the proteins (Figure 5B).

### Mutagenesis and enzymology

As expected, FADase inhibitor 4-(chloromercuri) benzoic acid significantly inhibited the activity of FADase, by ∼45%. To study the functional roles of key residues in the active domain, site-directed mutagenesis of Tyr21, Trp25, Tyr27 and Glu134 were carried out. These amino acids formed the catalytic center of FADase and interacted directly with substrates based on our crystal structure analyses (Figure 2,4). Mutations of the residues resulted in changes of both *Km* and *Vmax* (Table 2). Compared to wild-type FADase, the *Km* of E134A mutation increased by 1.163 mM and the *Vmax* of the E134A mutation decreased by 5.35 µ s^−1^. In contrast, the *Km* of the W25A mutation decreased by 1.628 mM and the *Vmax* of W25A mutation reduced by 9.44 µM s^−1^. Furthermore, the Y21A and Y27A mutations led to the complete loss of enzymatic activity.

**Table 2:** Enzymology analysis of FADase and mutants.

Mutants	*Km* (mM)	*Vmax* (µM/s)	*Kcat* (1/s)	*kcat*/*Km* (1/s*mM)
FADase	2.36	10.10	2.15	0.91
E134A	3.52	4.75	0.995	0.28
W25A	0.73	0.66	0.02	0.02
Y21A	ND*	ND	ND	ND
Y27A	ND	ND	ND	ND

*ND, no significant signal.

### The catalytic mechanism of FADase

**Figure 6:**
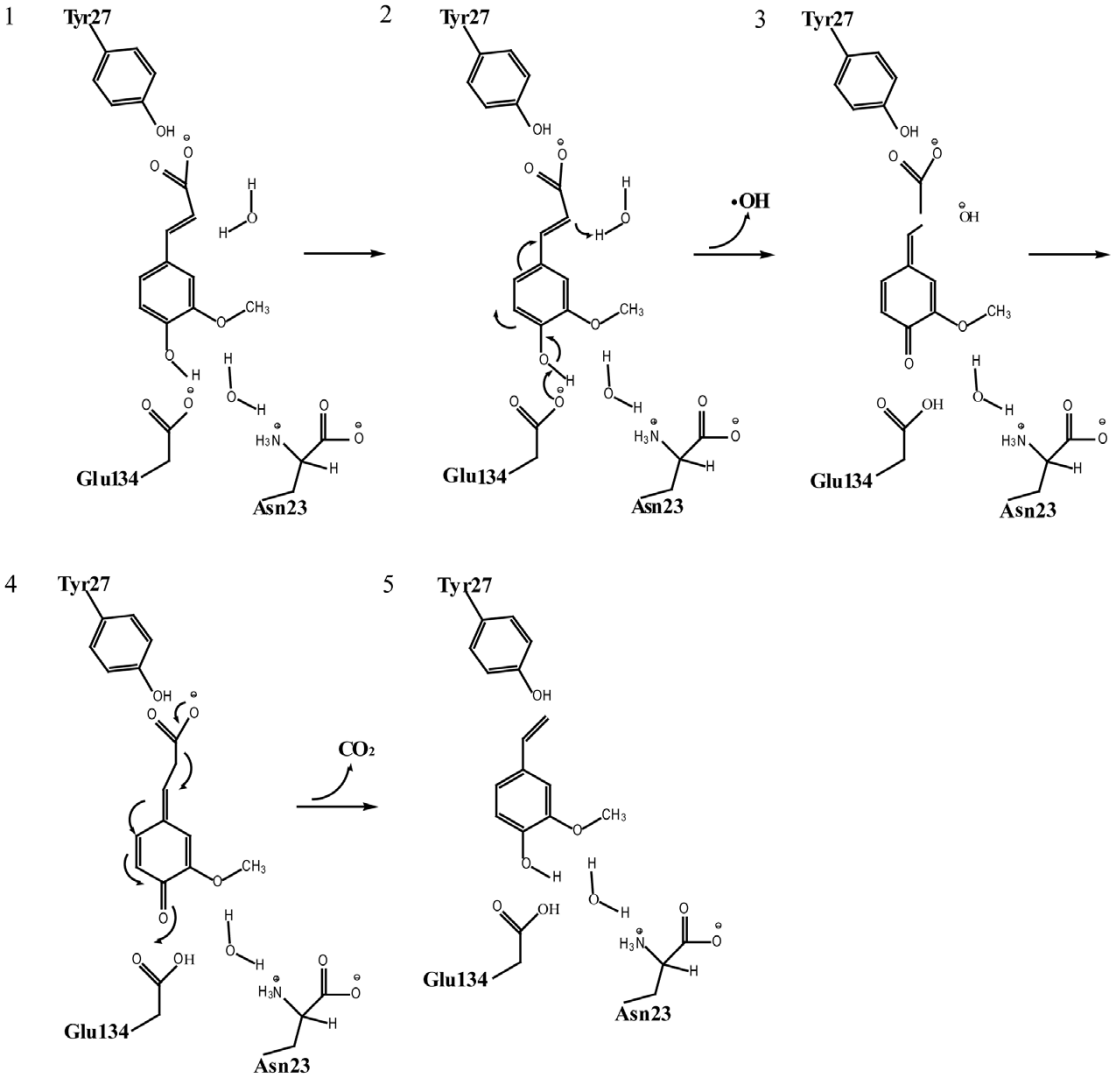
Proposed catalytic mechanism of decarboxylation by FADase. The proposed mechanism is based on inferences drawn from the structural and mutagenesis results presented here. 1. The substrate's hydroxyl group is deprotonated; 2. A nucleophilic center is formed at the ortho-carbon atom of the carboxyl group of ferulic acid. 3. A quinoid intermediate is formed. 4. The intermediate is cleft. 5. 4-vinylguaiacol is formed and CO_2_ is released.

Comparison of the crystal structures of pure FADase and FADase in complex with sodium ferulate revealed that an active hydrophobic pocket was formed when FADase crystals were soaked in a solution containing sodium ferulate. The combined structural and mutagenesis results enabled us to propose a two-step catalytic mechanism for decarboxylation of ferulic acid by FADase (Figure 6). In this proposal, Asn23 is more hydrophilic than the nonpolar amino acids because the amide group forms a hydrogen bond with water. Asn23 ensures the presence of a water molecule in the hydrophobic pocket. This water molecule interacts with the hydroxyl group of sodium ferulate and Glu134, creating a polar microenvironment. Under the action of Glu134's carboxylate anion, the substrate's hydroxyl group would be deprotonated to ensure the electron flow through ferulate. A nucleophilic center was then formed at the ortho-carbon atom of the carboxyl group of ferulic acid. The carbon moved towards the positive electric field formed by Tyr27 and the water molecule, forming a quinoid intermediate (Figure 6). Subsequently, the carboxyl of the quinoid intermediate started a second electron flow, and the electron-donating groups make the covalent bond heterolytic cleavage, producing 4-vinylguaiacol and releasing CO_2_.

## Discussion

In this study, a gene (*fad*) encoding the FADase enzyme was isolated from *Enterobacter* sp. Px6-4 and expressed in *E. coli* BL21 (DE3). Crystal structures of FADase and FADase in complex with a substrate analog sodium ferulate were characterized for the first time. The deduced amino acid sequence of the FADase from *Enterobacter* sp. Px6-4 showed 54–55% sequence identity to other decarboxylases from the other bacteria including *Bacillus amyloliquefaciens*, *Bacillus pumilus*, *B. subtilis* and *L. plantarum*, suggesting that FADase of *Enterobacter* sp. Px6-4 was also a member of the bacterial PAD family. Size exclusion chromatography showed that the recombinant FADase had a molecular mass of 44 kDa (data not shown), indicating that it was a homodimer consisting of two 23-kDa subunits, which was similar to other PAD from *B. pumilus* [36] and *Bacillus* sp. BP-7 [37]. From the analysis of predicted amino acid sequence of FADase, we found that there was no secretion signal sequence, suggesting that, similar to other decarboxylases, the FADase was an intracellular enzyme [36]. Interestingly, the highest amino acid sequence variability among the PADs from various bacteria was found in a region adjacent to the C-terminal portion of the proteins. This region had been suggested as responsible for enzyme-substrate specificity [38].

In the enzyme-substrate complexed crystal, we found that there was only one ferulic acid molecule in the two closely packed FADase monomers presented in the asymmetric unit (Figure 3). As the active site of one molecule faced the solvent side while the other blocked by the other monomer, the blocked active site could not bind the substrate (Figure 3A). Residues Ile41, Leu45, Val46, and Ile132 all contained aliphatic R groups and they are arranged close to each other in the three dimensional structure. The side chains of these residues tend to cluster together and their hydrophobic interactions stabilize the protein structure [39]. Residues Tyr21, Trp25, and Tyr27 contained aromatic R groups and their aromatic side chains are relatively hydrophobic. The aromatic R groups can participate in hydrophobic interactions, including attracting the substrate. These hydrophobic interactions play an important role in keeping the tertiary structure of the proteins [39]. Asn23 contains a polar uncharged R group and can form a hydrogen bond with a water molecule [39]. The Asn23 gets the polarity by its amide group. Glu134 has a negatively charged R group at pH 7.0 [39]. FADase undergoes conformational changes including the adjustment of spatial positions of functional groups during interaction with the substrate. The mutation from Glu134 to Ala134 in FADase resulted in the *Km* and *Vmax* reductions of 50.7% and 47.0%, respectively. These results suggested that though Glu134 played a very important role in proton transfer, it was unlikely the only proton donor. However, Glu134 might also assist the entry of the carboxylate group because of electrostatic repulsion which could destabilize the ground state of the substrate [40]. Based on the structure of FADase-substrate complex, we proposed that one water molecule was attached to the hydroxyl group of the substrate by hydrogen bonding and this bonding affected proton transfer. The mutation from Tyr27 to Ala27 resulted in an almost complete loss of the FADase activity. Tyr27 played an important role in attracting carboxylic acid to complete the decarboxylation step. The mutation from Tyr21 to Ala21 also abolished the enzymatic activity, suggesting that the substrate could not make optimal contacts with the active site. After the mutation of Tyr21 to Ala21, the pocket size was likely reduced and the substrate could not enter the active site. Trp25 acted as a lock to cover the active center. When Trp25 was mutated to Ala25, its lock function was abolished and the active pocket was exposed in the solution, facilitating the contact with the substrate. The decrease of *Km* in the W25A mutants suggested that this mutation increased the enzymatic affinity to ferulic acid. Compared to the wild-type FADase, the *Vmax* of W25A decreased by 93.5% of the wild type enzyme. These results suggested that Trp25 not only functioned in covering the active pocket, it also participated in catalyzing the transformation of ferulic acid.

The crystal structure, putative active site and proposed decarboxylation catalytic mechanism for PDC from *L. plantarum* (2W2A) were recently reported [23]. Both the FADase and 2W2A belonged to the PAD lyase family. After structure alignment between FADase and 2W2A, we observed that the half-opened bottom β-barrels were highly conserved and the main differences were in the N- and C-terminals. However, the active pocket of FADase was larger than that of 2W2A, suggesting that the FADase's active pocket could accommodate more substrate types. In addition, the active pocket of FADase was induced by the substrate while that of 2W2A could be formed without substrate induction. Because ferulic acid was toxic to many microorganisms, the formation of FADase active pocket to sequester ferulic acid could be an adaption mechanism for *Enterobacter* sp. Px6-4. When the active pockets of FADase and PDC were compared, several differences were apparent. First, while Tyr20, Tyr26, Ile40 and Val45 are located in the hydrophobic pocket of both FADase and 2W2A, residues Trp69, Val77 and Phe94 were not found in the active pocket of FADase. Second, while the residues of FADase involved in hydrogen bonding interactions with the substrate were Tyr27 and Glu134, in 2W2A, residues Tyr20 and Glu71 played corresponding roles. Third, in FADase, residues Trp25 and Asn23 played important roles as the “lock” and indirectly as proton donors, they were not found in the hydrophobic pocket of 2W2A. Therefore, further efforts are warranted to elucidate the relation between amino acid differences and catalytic mechanism of these enzymes.

## Supporting Information

Figure S1 **Cross-linking gel of FADase.** The dimer (∼43 kD) was detected. Molecular weight marker is labeled at the side of each lane. EGS: ethylene glycolbis.
(TIF)

## References

[1] Hartley RD, Ford C W, Lewis NG, Paice MG (1989). Phenolic constituents of plant-cell walls and wall biodegradability.. Plant cell wall polymers: biogenesis and biodegradation.

[2] Rosazza JP, Huang Z, Dostal L, Volm T, Rousseau B (1995). Review: biocatalytic transformations of ferulic acid: an abundant aromatic natural product.. J Ind Microbiol.

[3] Mathew S, Abraham TE (2006). Bioconversion of ferulic acid, an hydroxycinnamic acid.. Crit Rev Microbiol.

[4] Walton NJ, Mayer MJ, Narbad A (2003). Vanillin.. Phytochemistry.

[5] Priefert H, Rabenborst J, Steinbuchel A (2001). Biotechnological production of vanillin.. Appl Microbiol Biotechnol.

[6] Lomascolo A, Stentelaire C, Asther M, Lesage-Meessen L (1999). Basidiomycetes as new biotechnological tools to generate natural aromatic flavours for the food industry.. Trends Biotechnol.

[7] Muheim A, Lerch K (1999). Towards a high-yield conversion of ferulic acid to vanillin.. Appl Microbiol Biotechnol.

[8] Yoshimoto T, Samejima M, Hanyu N, Koma T (1990). Dioxygenase for styrene cleavage manufactured by *Pseudomonas*.. Japanese patent 2,195,871.

[9] Rabenhorst J, Hopp R (1991). Process for the preparation of vanillin.. U.S. patent 5,017,388.

[10] Washisu Y, Tetsushi A, Hashimoto N, Kanisawa T (1993). Manufacture of vanillin and related compounds with *Pseudomonas*.. Japanese patent 5,227,980.

[11] Labuda I, Goers SK, Keon KA (1992). Bioconversion process for the productuction of vanillin.. U.S. patent 5,128,25.

[12] Walton NJ, Narbad A, Faulds C, Williamson G (2000). Novel approaches to the biosynthesis of vanillin.. Curr Opin Biotechnol.

[13] Arfmann HA, Abraham W R (1989). Mircobial formation of substituted styrenes.. Z Naturforsch.

[14] Huang Z, Dostal L, Rosazza JPN (1993). Microbial transformations of ferulic acid by *Saccharomyces cerevisiae* and *Pseudomonas fluorescens*.. Appl Environ Microbiol.

[15] Huang Z, Dostal L, Rosazza JPN (1994). Mechanism of ferulic acid *Rhodotorula rubra*.. J Biol Chem.

[16] Nazareth S, Mavinkurve S (1986). Degradation of ferulic acid via 4-vinylguaiacol by *Fusarium solani* (Mart) Sacc.. Can J Microbiol.

[17] Rahouti M, Seigle-Murandi F, Steiman R, Eriksson K-E (1989). Metabolism of ferulic acid by *Paecilomyces variotti* and *Pestalotia palmarum*.. Appl Environ Microbiol.

[18] Degrassi G, DeLaureto PP, Bruschi CV (1995). Purification and characterization of ferulate and *p*-coumarate decarboxylase from *Bacillus pumilus*.. Appl Environ Microbiol.

[19] Cavin J-F, Barthelmebs L, Guzzo J, Van Beeumen J, Sannyn B (1997). Purification and characterization of an inducible *p*-coumaric acid decaroxylase from *Lactobacillus plantarum*.. FEMS Microbiol Lett.

[20] Cavin J-F, Barthelmebs L, Diviès C (1997). Molecular characterization of an inducible *p*-coumaric acid decaroxylase from *Lactobacillus plantarum*: gene cloning, transcriptional analysis, overexpression in *Escherichia coli*, purification and characterization.. Appl Environ Microbiol.

[21] Cavin J-F, Dartois V, Diviès C (1998). Gene cloning, transcriptional analysis, purification and characterization of phenolic acid decarboxylase from *Bacillus subtilis.*. Appl Environ Microbiol.

[22] Karmakar B, Vohra RM, Nandanwar H, Sharma P, Gupta KG (2000). Rapid degradation of ferulic acid via 4-vinylguaiacol and vanillin by newly isolated strain of *Bacillus coagulans*.. J Biotechnol.

[23] Rodríguez H, Angulo I, de Las Rivas B, Campillo N, Páez JA (2009). *p*-Coumaric acid decarboxylase from *Lactobacillus plantarum*: structural insights into the active site and decarboxylation catalytic mechanism.. Proteins.

[24] Li XM, Yang JK, Li X, Gu W, Huang JW (2008). The metabolism of ferulic acid via 4-vinylguaiacol to vanillin by *Enterobacter* sp. PX6-4 isolated from *Vanilla* root.. Process Biochem.

[25] Otwinowski Z, Minor W, Carter CW, Sweet RM (1997). Processing of X-ray diffraction data collected in oscillaction mode.. Macromolecular Crystallography part A.

[26] Matthews BW (1968). Solvent content of protein crystals.. J Mol Biol.

[27] McCoy AJ, Grosse-Kunstleve RW, Adams PD, Winn MD, Storoni LC (2007). Phaser crystallographic software.. J Appl Cryst.

[28] Emsley P, Cowtan K (2004). Coot: model-building tools for molecular graphics.. Acta Crystallogr D Biol Crystallog.

[29] Murshudov GN, Vagin AA, Dodson EJ (1997). Refinement of macromolecular structures by the maximum-likelihood method.. Acta Crystallogr D Biol Crystallogr.

[30] Laskowski RA, MacArthur MW, Moss DS, Thornton JM (1993). PROCHECK: a program to check the stereochemical quality of protein structures.. J Appl Cryst.

[31] DeLano W (2002). The PyMOL Molecular Graphics System, DeLano Scientific, Palo Alto, CA, USA..

[32] Bradford MM (1976). A rapid and sensitive method for quantitation of microgram quantities of protein utilizing the principle of protein-dye-binding.. Anal Biochem.

[33] Yang JK, Huang XW, Tian BY, Wang M, Niu QH (2005). Isolation and characterization of a serine protease from the nematophagous fungus, *Lecanicillium psalliotae,* displaying nematicidal activity.. Biotechnol Lett.

[34] Rao FV, Andersen OA, Vora KA, DeMartino JA, van Aalten DMF (2005). Methylxanthine drugs are chitinase inhibitors: Investigation of inhibition and binding modes.. Chem Biol.

[35] Nelson DL, Cox MM (2004). Chapter6 Enzymes..

[36] Zago A, Degrassi G, Bruschi CV (1995). Cloning, sequencing, and expression in *Escherichia coli* of the *Bacillus pumilus* gene for ferulic acid decarboxylase.. Appl Environ Microbiol.

[37] Prim N, Pastor FI, Diaz P (2001). Cloning and characterization of a bacterial cell-bound type B carboxylesterase from *Bacillus sp.* BP-7.. Curr Microbiol.

[38] Barthelmebs L, Divies C, Cavin JF (2001). Expression in *Escherichia coli* of native and chimeric phenolic acid decarboxylases with modified enzymatic activities and method for screening recombinan*t E. coli* strains expressing these enzymess.. Appl Environ Microbiol.

[39] Nelson DL, Cox MM (2004). Chapter3 Amino acid peptides and proteins..

[40] Cendron L, Berni R, Folli C, Ramazzina I, Percudani R (2007). The structure of 2-oxo-4-hydroxyl-4-carboxy-5-ureidoimidazoline decarboxylase provides insights into the mechanism of uric acid degradation.. J Biol Chem.

