# Supplementary figures and images for: Structural Basis of Enzymatic Activity for the Ferulic Acid Decarboxylase (FADase) from Enterobacter sp. Px6-4
Source: PLoS One. 2011 Jan 21;6(1):e16262. doi: 10.1371/journal.pone.0016262 (PMC3025021; doi:10.1371/journal.pone.0016262)

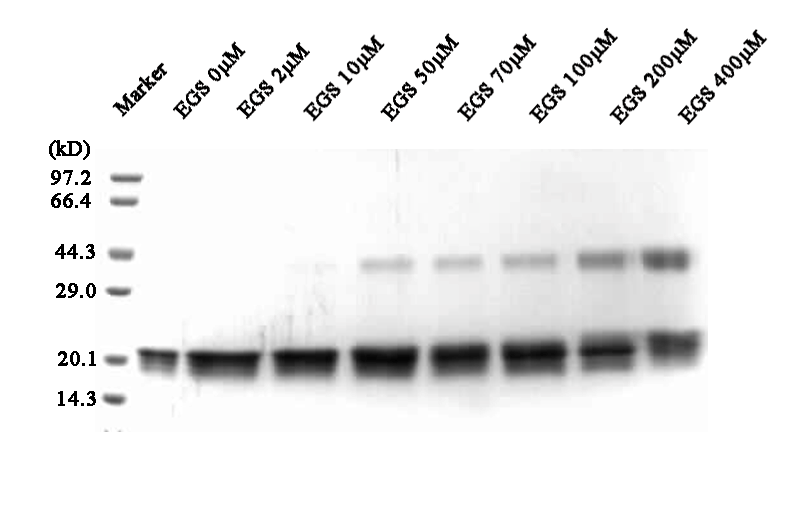

Supplement: Figure S1 — Cross-linking gel of FADase. The dimer (∼43 kD) was detected. Molecular weight marker is labeled at the side of each lane. EGS: ethylene glycolbis. (TIF) [file pone.0016262.s001.tif]
